# Dendritic Cells and Cancer Immunotherapy: The Adjuvant Effect

**DOI:** 10.3390/ijms222212339

**Published:** 2021-11-15

**Authors:** Sara Nava, Daniela Lisini, Simona Frigerio, Anna Bersano

**Affiliations:** Cell Therapy Production Unit, Neurology Unit, UCV, Neurological Institute “C. Besta” IRCCS Foundation, Via Celoria 11, 20133 Milan, Italy; daniela.lisini@istituto-besta.it (D.L.); simona.frigerio@istituto-besta.it (S.F.); anna.bersano@istituto-besta.it (A.B.)

**Keywords:** dendritic cells, immunotherapy, cancer, adjuvant therapy

## Abstract

Dendritic cells (DCs) are immune specialized cells playing a critical role in promoting immune response against antigens, and may represent important targets for therapeutic interventions in cancer. DCs can be stimulated ex vivo with pro-inflammatory molecules and loaded with tumor-specific antigen(s). Protocols describing the specific details of DCs vaccination manufacturing vary widely, but regardless of the employed protocol, the DCs vaccination safety and its ability to induce antitumor responses is clearly established. Many years of studies have focused on the ability of DCs to provide overall survival benefits at least for a selection of cancer patients. Lessons learned from early trials lead to the hypothesis that, to improve the efficacy of DCs-based immunotherapy, this should be combined with other treatments. Thus, the vaccine’s ultimate role may lie in the combinatorial approaches of DCs-based immunotherapy with chemotherapy and radiotherapy, more than in monotherapy. In this review, we address some key questions regarding the integration of DCs vaccination with multimodality therapy approaches for cancer treatment paradigms.

## 1. Introduction

Malignant growth depends on the failure of the immune system to recognize cancer cells, due to the ability of tumor cells to effectively silence the “danger signal” required for immunological activation. When the immune surveillance process fails, one of the protective mechanisms against tumor growth is lost. Strategies are being developed to correct this vaccination failure [1].

Since their discovery in 1973, Dendritic Cells (DCs) were considered the crucial Antigen-Presenting Cells (APCs) for the activation of the adaptive immune system and may represent important targets for therapeutic cancer interventions [2].

In mouse models, it was extensively demonstrated that DCs prepared for immunotherapeutic use can capture tumor antigens, which are released from tumor cells, and present them to T cells in tumor-draining lymph nodes. This DCs capability results in the generation of tumor-specific Cytotoxic T Lymphocytes (CTLs) that contribute to tumor rejection [3,4]. To achieve this, DCs need to be stimulated ex vivo with pro-inflammatory molecules and loaded with tumor-specific antigen(s). The goal of cancer vaccines is to elicit tumor-specific cell-mediated immune responses that will be sufficiently robust and long-lasting to generate durable tumor regression and/or eradication.

Since the 1990s, DCs were used in clinical trials for therapeutic vaccination of cancer patients. As natural DCs constitute only about 1% of peripheral blood mononuclear cells (PBMCs), several ways to generate DCs from precursors have been investigated for DCs’ vaccination purposes.

Natural circulating DCs or monocytes are isolated from autologous PBMCs obtained by apheresis. In case of monocytes, ex vivo differentiation into DCs are required. Both natural circulating DCs and monocyte-derived DCs are matured cells, as this is essential for effective T-cell activation [5]. DCs maturation is a complex process: maturation signals come from contact with pathogens or tissue injury in vivo or from a variety of stimuli in vitro; the outcome depends on the type of signals that the DCs receive.

During the vaccine manufacturing process, DCs are loaded with relevant tumor antigen(s) to induce a tumor-specific immune response in patients. Several methods to load DCs with antigen have been reported [6].

Protocols describing the specific details of DCs vaccination manufacturing vary widely. Differences cover all aspects of DCs vaccination, including culture methods, usage of DCs subsets, maturation strategies, antigen types and loading techniques. In addition, to obtain a highly effective product, the route of administration must also be considered.

Regardless of the employed protocol, side effects analyzed in the majority of DCs vaccination protocols resulted minimal, consisting mainly in flu-like symptoms, fever and local reactions in accordance with the injection site. Toxicity is extremely uncommon when DCs vaccination is given as monotherapy [7]. These data are confirmed by phase III trials where DCs vaccination is compared with placebo [8,9,10,11]. Therefore, DCs vaccination is considered safe for cancer patients.

A functional immune response is a complex and multi-step process. At least four components of the immune response are necessary for a positive result: the presence of appropriate APCs, the quality of induced CD4+ T helper cells, the control of regulatory T cells (Tregs) and the breakdown of the immunosuppressive tumor microenvironment. Thus, the ultimate role for vaccines may lie in the combination with other treatments. Clinical studies combining DCs vaccination with chemotherapy, radiotherapy (RT) and/or other targeted therapy have been performed, confirming, in Phase I, the safety of DCs [12,13,14,15].

**Figure 1:**
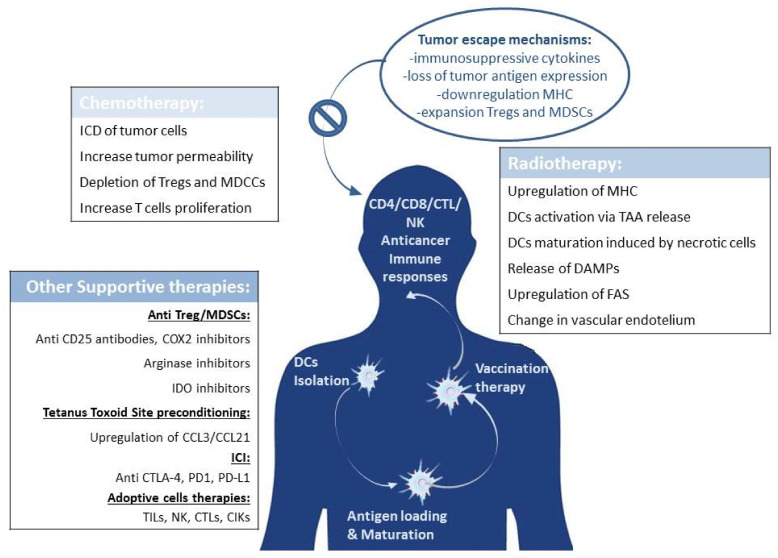
Combined therapy. Tumor escape mechanisms often inhibit the effect of dendritic cell vaccination. Conventional therapies such as chemo/radiotherapy can collaborate with DCs vaccination, promoting the activation of immune cells effectors and hampering the functions of immunosuppressive cells. Other innovative strategies are under investigation as a support to contrast tumor immune escape, such as inhibitors of Tregs and MDSCs; ICI, adoptive cells therapies and preconditioning of the pre-vaccine injection site.

Immunotherapy is a fast-moving field, and it is a major pathway of developmental therapeutics for metastatic patients. Lessons learned from early trials move to the hypothesis that combinatorial approaches of immunotherapy with DCs, chemotherapy, and RT rather than monotherapies can improve the efficacy of the cancer treatment option (Figure 1). The aim of this paper is to report an update review in the understanding of this synergy and in the potential advantages of a multimodal therapy.

## 2. Ex Vivo-Generated DC Vaccines

Natural DCs present in the human blood represents only about 1% of PBMCs. Despite the limited number, some authors have described the possibility of using natural DCs in clinical trials without an extensive culture period. Recently, it has been demonstrated that is possible to obtain more than 10 million plasmacitoid DCs, and an even higher number of BDCA-1 myeloid DCs, after a single leukapheresis, despite their low frequency among PBMCs [16]. Some clinical trials have proven the safety and feasibility of this “first generation” vaccine [17,18].

A major advantage of natural circulating DCs is their rapid isolation procedure with antibody-coated magnetic beads. Natural DCs subsets are postulated to be more powerful compared with monocyte derived DCs due to their unique functional properties and crosstalk capacity [19].

To overcome the limit of natural DCs low number in the peripheral blood, starting from 1994 investigators have sought a way to generate, ex vivo, large amounts of DCs, starting from the CD14+ cell subset from apheresis, with the aim of using them as “second generation” vaccines for patient treatment [20,21]. Monocytes require an ex vivo differentiation and maturation process to originate DCs.

### 2.1. Maturation Protocols

As mentioned above, ex vivo generation of a large amount of DCs can occur using different culture protocols. After isolation, CD14+ cells are cultured with cytokines, typically granulocyte-macrophage-colony stimulating factor (GM-CSF) and interleukin (IL)-4 for four to five days, after which DCs display an immature phenotype.

Historically, in the first clinical studies involving the use of DCs for patient treatment, the authors planned to use immature or semi-immature DCs-derived monocytes. Later trials have demonstrated that mature DCs use is better in terms of enhanced migratory capacity, immunogenicity, expression of HLA-DR and costimulatory molecules. These findings are strongly related to an improved clinical outcome [21,22]. Taken together, these conclusions show the superiority of mature DCs in antigen presence and therefore in inducing T-cell responses [22,23,24].

The choice to use mature DCs in clinical trials is closely related to the optimization of the ex vivo DCs culture protocol, mainly in terms of the maturation cocktail. In vivo, the contact with the pathogens or tissue injury starts the DCs maturation process; the outcome depends on the type of signals that the DCs receive. Ex vivo DCs maturation depends on a variety of stimuli to which DCs are submitted during the culture process. The best DCs preparation protocol should give origin to DCs with a high migration capacity towards lymph nodes, DCs able to present antigens and co-stimulation to T cells, and finally DCs able of surviving to T-cell activation.

The most widely used DCs maturation cocktail includes cytokines GM-CSF and IL-4 and tumor necrosis factor-α (TNFα) in combination with IL-1β, IL-6, prostaglandin E2 (PGE2) or monocyte-conditioned medium [21,25]. This mix produces mature DCs with a superior ability to stimulate T cells than immature DCs [26] and an improved migratory capacity to mobilize DCs towards lymph nodes where they can prime T cells [27]. However, there are data supporting the fact that mature DCs preferentially recruit Tregs, thus potentially dampening any immune response initiated.

Some authors also theorized the addition of Interferon γ (INFγ), Poli-IC and Toll Like Receptors (TLR) ligands or costimulatory pathways (CD40-CD40L) to the maturation cocktail, thus resulting in the production of high levels of IL-12, which directs a Th1-type T cell response [28,29,30,31]. In clinical trials, Poly-IC matured DCs vaccines are reported as well-tolerated, producing immunological and clinical responses. However, there are no clinical trials that directly compare Poly-IC matured DCs with cytokine cocktail matured DCs. CD40L induced CD86 and CD83 expression on DCs, but it seems that it did not improve anti-tumor specific T cell proliferation. The use of CD40L was compared to a cytokine cocktail and no difference was found in the immunological response [32].

Some authors showed that DCs may also be activated by electroporation with mRNA encoding constitutively active TLR4 and CD40 ligand, obtaining DCs able to suppress Tregs functions and to reprogram Tregs to Th1 cells under certain circumstances [33,34].

### 2.2. Extensive-vs. Short-Culture Period

An important aspect in terms of DCs preparation for clinical use is the time requested for cell manufacturing. Traditional protocols have a duration of seven to eight days and foresee medium and repeated cytokines addition. In a good manufacturing practice (GMP)-compliant context, this promotes increased costs in terms of consumables, as well as operator activity in a clean room. Some groups have demonstrated that it is possible to obtain DCs with the safety and efficacy requested by regulatory agencies using short culture protocols in three to five days [35,36].

Together to the shortening of the culture, in the last years manufacturing challenges also concerned the transfer from culture flasks to bags and, finally, bioreactors. This constitutes a great advantage in terms of GMP requirements and allows an easier and safer passage from research DCs preparation protocols to automatic DCs preparation of commercial drugs. Manufacturing cells to be used as drugs for patient treatment were traditionally prepared in “open” polystyrene-based vessels both for adherent and suspension cell cultures. Open cell culture systems require the opening of flasks or plates for media changes and other cell culture manipulations [36,37]. To decrease the risk of product contamination by individual operators and to facilitate the scale-up or scale-out and automation of cell production, “closed” culture systems are thus generally preferred by regulatory authorities to comply with current good manufacturing standards.

### 2.3. Tumor Antigens and DCs Loading

Antigen processing and presentation is a biological mechanism used by APCs to capture, process and expose the antigen on the cell surface in association with the histocompatibility complex. DCs are specialized APCs effective for carrying out this role, since they are able, once the antigen has been captured, to move into the lymph nodes and to present it to T lymphocytes activating them. For this capability, the practical use of DCs as delivery vehicles for antigen presenting finds extensive application in the immunotherapeutic field for a wide range of tumor types.

To date, several methods have been evaluated for loading DCs with tumor antigen in antitumor immunity induction. However, the best approach for loading DCs to induce the strongest CTLs responses has not yet been identified [38].

Tumor cell lysates are the most used source to load DCs. The lysis procedure mostly assures that tumor cells die before antigen loading, thus guaranteeing the safety of these cells. Freeze-thaw processing, UVB irradiation to induce cellular necrosis and apoptosis, manual and/or mechanical breakdown of the tumor, and whole tumor Hypoclorous acid Oxidation (HOCl) are the most common procedures to prepare tumor lysates [39,40,41,42,43]. Loading DCs with tumor lysates has the advantage off reducing the possibility of escape tumors from immunological control, bypassing the need to identify specific antigens, which in many cases have not yet been identified, allowing this methodology to be applied to a wider range of tumor types. Furthermore, targeting a full range of antigens has the advantage of preventing antigen-loss tumor variants [44]. The main disadvantage in the use of whole tumor cell lysates is the possible presence of normal tissue antigens that can induce autoimmune processes [45].

Fusion of DCs with tumor cells is another strategy to deliver all tumor cell antigens onto DCs to generate a specific CTL immune response [46].

DCs loading with peptide(s) is an alternative modality to induce antitumor responses. Peptides with defined epitopes have the advantage of inducing epitope-specific immune response limiting possible autoimmune reaction. However, this approach can limit the studies only to those tumors for which specific antigens have been identified. Furthermore, the same epitope can induce different individual reactivity and limit the application to MHC-matched patients only. In addition, the possibility of tumor escape from immune recognition is higher than using a tumor antigens broad spectrum. To overcome this limitation, loading DCs with whole protein may be preferred.

Liposome-mediated fusion, electroporation, and osmotic loading are the most popular methods for loading DCs with protein [42]. The use of DNA and RNA constructs is a new innovative method for DCs antigen loading. Transfection with DNA and RNA has been tested and has a lot of advantages: (a) generation of large amount of immunogenic DNA/RNA to be used in clinical application, even when tumor tissue is limited and/or difficult to be obtained; (b) increased efficiency of the immune response induction; (c) nucleic acid-based constructs involve synthesis of antigens that can be expressed, processed and presented in a complex with MHC molecules for a long time and efficiently, inducing T cells immune response [38,42,45]. In addition, RNA sequencing technologies to determine somatic mutations inside the tumor is an evolving technique that allows for the release of the neo-antigens, and possibly enables patient and tumor specific antigens for vaccines. However, limits are due to the frequency of neo-antigens strongly dependent on the tumor type [1].

The described methods for loading tumor antigens are generally valid; but due to the different nature of tumor cells and their variety, a method may be more suitable for some types of tumors but not for others, in this way provoking tumor immune responses of different efficiency [38,45]. Identifying the optimal DCs loading strategy for each tumor type therefore represents a challenge for cancer immunotherapy.

### 2.4. Clinical Trials

The therapeutic use of cancer vaccines has recently been reviewed as a consequence of clinical trials series that have yielded encouraging clinical outcomes. First, treatment of metastatic prostate cancer with sipuleucel-T (also known as APC 8015), a cell product based on enriched blood APCs that are briefly cultured with a fusion protein of prostatic acid phosphatase (PAP) and GM-CSF with the result of an approximately a four month longer median survival in Phase III trials [47,48]. Sipuleucel-T has been approved by the US Food and Drug Administration (FDA) for metastatic prostate treatment and cancer resolution, thereby paving clinical development and regulatory paths for the next generation of cellular immunotherapy products. Second, a Phase III trial in metastatic melanoma that tested the peptide vaccine in combination with high-dose IL-2 versus IL-2 alone, showed significant improvement in overall response rates and progression-free survival in patients who received the vaccine [49]. Third, a Phase III trial in patients with follicular lymphoma showed that idiotype vaccine therapy significantly prolongs duration of chemotherapy-induced remission [50]. Furthermore, a randomized Phase II trial of a poxvirus-based vaccine (PROSTVAC) targeting men with Prostate Specific Antigen (PSA) in metastatic castration-resistant prostate cancer showed an improved overall survival in patients when compared with the ones receiving control vectors (an observed difference of 8.5 months in median survival) [51]. DCs generated ex vivo by culturing haematopoietic progenitor cells or monocytes with cytokine combinations have been tested as therapeutic vaccines in cancer patients for more than a decade [52]. These clinical studies concluded that DCs-based vaccines are safe and can induce the expansion of circulating CD4+ T and CD8+ T cells which are specific for tumor antigens. Objective clinical responses have been observed in some patients. Clinical response takes time to develop, but remissions can be very long-lasting [7,53]. Tumor antigens selection for loading DCs is an important parameter. Candidate tumor antigens include unique (mutated) antigens and shared non mutated self-antigens [54,55,56,57]. To generate broadly applicable vaccines, non-mutated self-antigens have often been selected. These, however, have potential shortcomings: the range of high-avidity clones might be depleted through negative selection [58,59], and the existing memory T cells often include Treg cells. Using mutated antigens might avoid these drawbacks. Cancer vaccines designed to elicit strong immune responses against these mutated antigens will require a fully personalized approach. A few years ago this represented a considerable challenge; fortunately, the development and application of RNA sequencing (RNA–Seq) technologies led to the determining of the complete range of mutated antigens from patients’ primary tumor and metastases, thereby allowing patient customized therapeutic vaccines.

## 3. Immunotherapy in Combination with the Standards of Care: May It Help?

Multiple clinical trials have investigated the role of immunotherapy combined with radio/chemotherapy in cancers (Table 1). Mixed approaches can promote and enhance the immunogenic environment through increased antigen presence, phagocytosis, cell death, and immune-mediated tumor surveillance [60]. Early trials have shown promising efficacy and tolerable adverse effects with multimodality therapy; the combination of conventional therapy with immunotherapy seems to result in the enhancement of T cell activation and function, depletion of Tregs, and reversion of T cell exhaustion and anergy.

### 3.1. DCs Immunotherapy Combined with Chemotherapy

The combination of DCs with chemotherapy seems unusual, as chemotherapy is known to have an immunosuppressive effect: cytotoxic chemotherapy induced depletion of leukocytes due to non-specific cell death of proliferating cells [61,62,63] is presumed to occur through a non-inflammatory apoptotic process or by induction of immune tolerance [61,62,63]. Chemotherapy could therefore theoretically reduce the efficacy of immunotherapy due to the depletion of the peripheral pool of available immune cells and induced tolerance. However, new evidence has led to a reevaluation of these conclusions.

Recent studies have indeed shown that chemotherapy also has out of target immunological effects, like immunogenic cell death (ICD) of tumor cells, which cause enhancement of anti-tumor immunity [64,65], and increasing tumor cell permeability to CD8+ T-cell–derived cytolytic factors. Moreover, this strategy has several others immune-potentiating effects: for example, depletion of myeloid-derived suppressor cells (MDSCs) and Tregs.

Chemotherapy creates a cytokine milieu for optimal expansion of antitumor effector cells; for this reason, chemotherapy has been applied in combination with adoptive T-cell transfer [66,67], with the result of a synergistic activity of the two treatments [68,69,70].

Many chemotherapeutic drugs, such as cyclophosphamide, temozolomide (TMZ), and gemcitabine seem to be the cause of tumor cell death by anti-tumor immunity [71].

Administration of cyclophosphamide prior to DCs therapy was found to prolong survival in mouse models of mesothelioma, melanoma and colon carcinoma compared to monotherapy. This appears associated with a decrease in Tregs and a subsequent increase in T cells.

TMZ is an alkylating agent mainly used to treat glioblastoma (GBM) and melanoma due to its ability to cross the blood brain barrier. TMZ at high doses induces lympho-ablation while at low doses it mainly targets Tregs. Administration of TMZ prior to DCs therapy in patients with melanoma reduced circulating Tregs: studies indicate that administration of TMZ before or during DCs therapy could strengthen the efficacy of DCs therapy, while DCs therapy followed by TMZ may adversely affect DCs-induced antitumor immunity [71]. Some data in GBM suggest that TMZ-induced lymphopenia combined with anti-EGFRvIII peptide vaccination may reduce helper of T cells fraction and enhance humoral responses [72]; other preclinical data indicate that TMZ may impair the antitumor activity of CD8+ T cells [73,74,75,76]. Eoli et al. suggest that systemic administration of TMZ in standard treatment can limit the antitumor immune response of CD8+ T cells induced by DCs vaccine and their long-lasting response [77]. Interestingly, preclinical data have recently indicated that standard, but not metronomic, doses of TMZ increased exhaustion markers in tumor infiltrating lymphocytes [78].

In 2008, Heimberger et al. suggested that chemotherapy is not necessarily counterproductive to immunotherapy as long as it is administered outside the therapeutic window, during which vaccine induced CTLs are more reactive and active against tumors [79]. Outside this effector phase, TMZ lympho-depleting effects may indeed be desirable, as they can reduce immunosuppressive lymphocytes such as Tregs and thus improve the local immune profile [79,80].

Administration of gemcitabine improves antitumor immunity by depleting MDSCs and Tregs. In mouse models of colon and pancreatic cancer, gemcitabine treatment before and after DCs therapy prolonged survival compared to untreated mice at greater extent than monotherapies. In a mouse pancreatic model, simultaneous treatment of DCs therapy and gemcitabine delayed tumor growth and prolonged survival in comparison with both monotherapies [64]. DCs-based chemo-immunotherapy is about to become an important treatment model in cancer immunotherapy [81].

So far, no clinical studies have been reported that exclusively tested the combination of chemotherapy and DCs vaccination. In melanoma, a phase III study shows encouraging data following combined treatment with chemotherapy and DCs vaccination with the addition of a Cyclooxygenase-2 (COX-2) inhibitor, and in lung cancer with the addition of autologous T cells. The two randomized trials show a longer overall survival than the one deriving from chemotherapy alone [82,83,84].

### 3.2. DCs Therapy Combined with Radiotherapy

RT is commonly considered an immunosuppressive agent that non-selectively affects rapidly dividing cells [85,86]. In particular, T lymphocytes prove to be extremely sensitive to ionizing radiation [87]; therefore, it is natural to assume that RT is counterproductive to immunotherapy, as it systematically eliminates antitumor response key mediators.

However, several studies have suggested that the immune system has an important role with regard to radiation’s therapeutic effects, promoting tumor cell death in the radiation area; for all these reasons, combining radiotherapy with DCs therapy may give origin to synergistic effects and extend clinical reactions. Stone and colleagues were among the first to demonstrate the involvement of the immune system in radiation’s therapeutic effects [88]. In a chemically induced fibrosarcoma model, it was calculated that the radiation dose needed to control 50% of tumor cells. After the immune system stimulation through a crude bacterial preparation, the dose of radiation required to control tumors was significantly reduced [88]. Subsequent studies have shown that the immune cells most involved in this mechanism are CD8+ [89,90,91,92]. Considering these interactions, the combination of radiation with immunotherapy could increase radiosensitization and improve local tumor control.

One of the best-studied mechanisms through which radiation can enhance immune responses or immunotherapy efficacy is the upregulation of class I MHC [89,93,94,95,96,97]. Since many cancers downregulate MHC expression as a mechanism to escape immune system detection, this upregulation is an important component of immune response. In GBM, for example, MHC downregulation is a well-described phenomenon [98,99]. In the treatment of this tumor, RT increases the expression of MHC molecules, thereby counteracting a major strategy for immune evasion from GBM. In the murine model of glioma GL261, Newcomb et al. observed that whole-body radiotherapy (WBRT) was able to induce upregulation of β2-microglobulin light chain subunits of the MHCI complex on glioma cells, with a concomitant increase of CTLs and infiltration of helper T cells. Furthermore, administration of both WBRT and allogeneic GL261 vaccination resulted in a survival advantage over WBRT alone, with superior long-term survival [100]. The increase in antigen presentation following MHC overexpression after RT has the advantage of providing a natural target for vaccine-induced antitumor immunity.

Radiation also appears to activate DCs and enhance tumor cross-presentation by causing the release of tumor antigens via inflammatory cell death, DCs activation, migration and cross-presentation of tumor antigens resulting in tumor-specific T cells activation and proliferation [101]. It has been shown that exposure to necrotic cells induces DCs maturation and a better antitumor response of the host secondary to cross-priming with effector lymphocytes [102,103]. These findings are the key steps through which radiation can induce antigen-specific systemic anticancer immune responses.

As radiation therapy induces the death of immunogenic cells, it causes the release of damage-associated molecular patterns and tumor-derived antigens, leading to initiation and migration of DCs towards lymph nodes and induction of systemic antitumor immune responses [104,105,106]. The survival benefits of combined RT and DCs vaccination have been described in several tumor models, including melanoma [107], prostate cancer [108], liver metastases [109] and breast cancers [110], and also in multiple preclinical models [111,112,113,114] in which decreased tumor mass and prolonged survival compared to monotherapy were observed. For example, intra-tumor injection of DCs following 15Gyofexternal beam RT (EBRT) in a mouse model enhanced tumor specific CTLs activity and effective antitumor immunity not observed with either method alone. Induction of systemic immunity was observed in a squamous cell carcinoma mouse model, where the combination of radiotherapy with intratumoral administration of DCs increased the number of CTLs in the tumor-draining lymph node rather than DCs monotherapy [111].

Many other immunostimulatory mechanisms have emerged from studies on the effect of RT. Among these, the upregulation of FAS represents an interaction mechanism between radiation and the immune system [93,115,116] expressing FAS-L, which may or may not be specific for tumor-associated antigens.

Other mechanisms concern changes in the vascular endothelium that enhance extravasation of immune cells [117] and increased expression of chemokine attractants that enhance immune cell migration and invasion [118,119]. Many groups have confirmed an increase in tumor infiltrating lymphocytes (TILs) in experimental models after irradiation [89,93,115,120,121,122,123].

### 3.3. Cryoablation

Among therapies, cryoablation should also be mentioned.

Cryoablative therapy is a process that uses extreme cold to destroy tissue. Ablation occurs in tissue that has been frozen by at least three mechanisms: (a) the formation of ice crystals which disrupt cell membranes; (b) the coagulation of blood, thereby interrupting blood flow; and (c) the induction of apoptosis. Cryoablation is mostly indicated for tumors at an early stage or those not eligible for surgery. The synergies of local ablative techniques together with systemic treatments are currently under investigation in interventional oncology. Given that cryoablation provides a pool of antigens visible for the immune system to induce an immune-specific activation directed against the tumor cells, it could be a very useful adjuvant anticancer therapy [124].

## 4. Combating Tumor Immune Evasion

DCs vaccination may be more efficient in combination with therapies that break the suppressive tumor microenvironment. Tumor escape mechanisms are different and include: the secretion of immunosuppressive cytokines and activation of negative regulatory pathways, the loss of tumor antigen expression or downregulation of class I MHC expression with impaired immune recognition, and the expansion and recruitment of Treg and MDSCs [125]. Furthermore, the tumor vascularity itself can form an important barrier for T cells to reach the tumor [126]. Some mechanisms highlighted by combinatorial therapies appear to be able to counteract tumor escape mechanisms and make DCs-based immunotherapy more effective: but so far only a few have been studied in clinical trials and may not be sufficient or not always an effective treatment. Many mechanisms to counter immune escape have been studied. The use of anti-CD25 antibodies is able to deplete Tregs, demonstrating better immune-mediated tumor rejection in mouse models [127]. Unfortunately, the same approach is not valid in humans despite the efficient depletion of Tregs from the peripheral circulation and the increased frequencies of tumor-specific T cells [128,129]. Conversely, Treg-lowering drugs appear to have paradoxical immunological effects that could compromise DCs vaccination activity as natural killer (NK) cell depletion and induction of tolerogenic DCs [130]. Together with Tregs, MDSCs directly suppress CD8+ T cell responses at the tumor site, hindering the immune response and supporting tumor growth. To target MDSCs, several interventions are being studied, including COX-2 inhibitors and arginase inhibitors [131]. Furthermore, inhibitors of the Indoleamine 2,3-Dioxygenase (IDO) pathway form a new class of immunomodulators that inactivate Tregs and MDSCs with unknown substantial immunological effects that compromise any effective antitumor response [132]. The combination of an IDO inhibitor and DCs vaccination is currently being tested during phase II studies in prostate and breast cancer (NCT01560923; NCT01042535-phase II), and is so far proving to be tolerated.

### 4.1. Dendritic Cells Combined with Immune Checkpoint Inhibition

It is well known that tumor cells are able to upregulate the expression of checkpoint molecules (such as CTLA-4, PD-L1, and PD-1), leading to anergy of cytotoxic T cells in the tumor microenvironment [133]. Immune checkpoint inhibitors (ICI) antagonize these molecules and thus aim to increase the antitumor immune response. In 2010, ipilimumab (a monoclonal antibody targeted to CTLA-4) was shown to provide clinical benefit in cancer patients (melanoma and other tumors), extending median overall survival (OS) to 10 months [134]. In 2014, two other monoclonal antibodies (pembrolizumab and nivolumab) were approved for targeting the PD-1 pathway in the treatment of metastatic melanoma, and these were followed by the anti PD-L1 antibodies avelumab, atezolimumab and durvalumab [135,136,137]. Other checkpoint molecules such as TIM-3 and LAG-3, currently in various stages of clinical investigation, have been shown to inhibit antitumor immune response [138].

Despite promising results, ICI also showed limitations: PD-1 inhibitors have an objective response rate ranging from 50% to 80% in melanoma, Merkel cell carcinoma and carcinoma squamous cells; an average of 15–30% in most other cancers; while virtually no improvements have been seen in cancers such as pancreatic cancer [139]. Combining PD-1 blockers with other ICI may improve the response rate, but with unacceptable increased toxicity related to immune adverse events. Since ICI requires pre-existing anticancer T cells at the tumor site and their clinical efficacy depends on the extent of T cell infiltration [140], their combination with anticancer vaccines is an obvious strategy to be pursued in order to raise awareness. Both ICI and DCs vaccination exert their effects primarily through modulation of the immune system acting in this way at different stages of the cancer immune cycle. For the ICI response, tumor-specific T cells must be present in the tumor microenvironment, the generation of which can be aided by DCs vaccination [141]; a higher number of tumor-infiltrating lymphocytes is associated with a better response to ICI. In contrast, DCs vaccination-induced T cells are often hampered by the immunosuppressive tumor environment. ICI could help the effector functions of these T cells by reducing inhibition through PD-1 signaling or by enhancing T cell activation through CTLA-4 modulation.

However, contrary to preclinical data, clinical data on combined treatment with ICI and DCs vaccination in humans are still limited. In 2009, Ribas et al. reported the safety of the combination of tremelimumab (CTLA-4 mAb) and DCs vaccination in patients with melanoma [142] in which four of 16 patients (25%) achieved an objective clinical response. Moreover, Wilgenhof et al. showed an overall survival of 38% in 39 patients with metastatic melanoma treated with a combination of ipilimumab and DCs vaccination [143]. Clinical trials suggest little further toxicity from adding DCs vaccines to ICI. An important aspect of the combined strategies is the timing of administration [144]. It would seem logical to first administer DCs vaccines to generate tumor-specific T cells and subsequently release immunosuppression with anti-PD-1 mAb. In contrast, the timing of administration of DCs and ipilimumab vaccines may be more complex, since both ipilimumab and these vaccines exert their functions in the priming phase of T cells. Indeed, in a preclinical model of prostate cancer, an optimal response to ipilimumab was shown when given on the same vaccination day [145].

### 4.2. Dendritic Cells Vaccination Combined with Adoptive Cell Therapies

In recent years, the rapid development of cancer immunotherapy, such as TILs, NK, CTLs, cytokines, induced killer cells (CIKs) and other immune cells, has provided a novel approach to a cancer treatment considered as a fourth-line cancer therapy [146]. Among these, CIKs alone or in combination with DCs have attracted increasing attention as an effective cellular immunotherapy [147,148]. CIKs, which are cytotoxic lymphocytes generated by the incubation of peripheral lymphocytes with anti-CD3 monoclonal antibody, interferon (IFN)-γ and interleukin (IL)-2, consist mainly of the CD3+ CD56+ subset. CIKs induce apoptosis of tumor cells and kill them through direct contact and cytokines secretion such as IL-2 and IFN-γ. The combination of DCs and CIK leads to a notable increase in cytotoxic activity [147]. Several studies have indicated that both CIKs and DC-CIKs were effective in treating multiple solid cancers, including non-small cell cancers of the lung, breast, colon and other types, without serious adverse reactions [147,149].

Studies have shown that CIKs/DC-CIKs combined with different chemotherapy regimens for the treatment of gastric cancer shows better efficacy than that shown by treatment with chemotherapy alone. However, clinical trials of CIKs/DC-CIKs cellular immunotherapy are still in the initial phase of study.

With the help of DCs, CIK cells could eradicate the malignant cells in three ways: direct disintegration of malignant cells; induction of malignant cells apoptosis by secreting several cytokines; direct regulation of immune response by killing malignant cells. Interestingly, DC-CIKs immunization also generates long-lived CD8+ T cells, suggesting DC-CIKs immunotherapy may be effective to prevent cancer relapse [150].

Also NKT cells exhibit several desirable anticancer properties due to their ability to directly lysate cancer cells. These cells secrete both TH1 and TH2 cytokines which can modulate T cell activity and promote DCs maturation through CD40L/CD40 mediated interactions [151]. The synergistic interaction between NKT and DCs cells leads to increased activation of CD4+ and CD8+ T cells and has been effective in inducing strong and long-lasting immune responses. In one study, the authors pulsed DCs with alpha-galactosylceramide, an activator of NKT cells, and DCs were then co-cultured with NKT cells isolated from glioma patients. NKT cells showed robust expansion and functionality with a significant increase in IFN-γ secretion. Furthermore, expanded NKT cells demonstrated significant ex vivo cytotoxic activity against U251 glioma cells [152]. Liu et al. also highlighted the efficacy of NKT cells as a DCs adjuvant against gliomas using pulsed DCs with glioma-derived exosomes and galactosylceramide in orthotopically implanted GBM cells. The authors showed an increased survival response compared to pulsed DCs with tumor lysate or galactosylceramide alone [153]. Therefore, by optimizing the pulsed antigen for DCs using glioma-derived exosomes, instead of the entire tumor lysate, through the addition of an adjuvant in the form of NKT cells, Liu et al. have shown that combinatorial therapy can lead to synergistic cytotoxic antitumor activity against gliomas.

## 5. How to Further Improve the DCs Vaccine Efficacy: Lessons Learned from Site Preconditioning in Glioblastoma

DCs vaccines have so far shown limited efficacy in tumor immunotherapy. Factors that could contribute to their ineffectiveness are not well understood even if numerous attempts have been made to improve their efficiency. Previous studies involving DCs vaccine injections have shown that only a small fraction of DCs actually reach lymph nodes, possibly explaining why many patients do not respond well to therapy.

Among studies, Mitchell et al. found that priming the vaccine site with a booster antigen improves DCs vaccines efficacy in patients with GBM [154].

The authors conducted a randomized, blinded study in which 12 patients received vaccine site preconditioning through Tetanus/diphtheria (Td) Toxoid, a potent booster antigen. Patients who received TdT had significantly more antigen specific DCs in the drainage lymph nodes of the vaccine site than patients who received DCs alone. Patients who received TdT also had a significant increase in progression-free and overall survival, suggesting that DCs increased migration to vaccine site-draining lymph nodes is correlated to improved progression-free and overall survival.

To investigate the mechanism of this behavior, the authors observed that serum levels of chemokine (motif C-C) ligand 3 (CCL3) were increased in the patients. Since virtually all people have been vaccinated against tetanus, the booster antigen can locally attract CD4 + T cells that release the CCL3 chemokine, upregulating CCL21 expression.

These assumptions have recently been confirmed by Eoli and colleagues [77]. In a phase I-II clinical study (DENDR2), 12 patients were treated with DCs vaccinations combined with dose-dense TMZ. Subsequently, in eight patients defined as Variant (V)-DENDR2, the vaccine site was preconditioned through Tetanus Toxoid (TT) 24 h before DCs vaccination and TMZ was avoided. Four of 12 DENDR2 patients reached OS9, five of eight V-DENDR2 patients (62%) reached OS9, and one patient OS >30 months. A robust CD8+ T-cell activation and memory T-cell formation were observed in V-DENDR2 OS > 9. The authors concluded that TT vaccine site preconditioning and lack of TMZ could contribute to the efficacy of DCs immunotherapy by inducing an effector response, memory, and helper T-cell generation.

Taken together, these data suggest that DCs vaccine responses in GBM patients could be improved through TdT preconditioning, and further proposing that DCs migration to VDLN could serve as a biomarker to predict patient response. Mitchell and colleagues speculated that TdT injection would work by inducing local inflammation at the vaccine site, and were surprised to see a systemic effect [154]. Increased DCs migration to lymph nodes occurred on both sides of the body, even if patients received the injection only on one side. Preconditioning with a toxoid to enhance tumor-specific immune responses has potential implications for of many types of cancer treatment.

## 6. Rationale for DC Vaccination in the Adjuvant Treatment of Cancer

Surgical resection with curative intent aims at removing, in the oncological patient, all the tumor burden, although often residual occult disease remains and can eventually lead to relapses [155].

The application of adjuvant treatment such as DCs-based immunotherapy with the aim of killing cancer cells might reduce relapse chances.

It is becoming increasingly clear that higher tumor burden is associated to higher tumor-induced immune suppression: several soluble elements secreted by tumor cells are known to suppress infiltrated effector T cells [156,157,158]. Tumors are able to upregulate IDO by inhibiting effector T cells [159] and Tregs and MDSCs are capable of inducing anergy in T cells [160]. Immune suppression associated with tumor burden is generally considered to be the underlying cause of low clinical response to DCs vaccination, as monotherapy, in metastatic disease [161]. The integration of immunotherapy within the standard postoperative therapy for patients is based on the presumed mutually beneficial effect of conventional treatment strategies and immunotherapy.

Adjuvant DCs vaccination has been extensively studied in GBM, melanoma and other tumors. GBM is commonly treated with maximally safe surgery and adjuvant temozolomide (TMZ) combined with radiotherapy, which is the so-called Stupp protocol [162]. In contrast to most malignancies, distant cerebral metastases seldom occur in GBM. However, even with extensive treatment, residual disease invariably remains, and recurrence is certain, with patients having a median survival of ~15months [163]. A similar effect as reported for GBM trials has also been retrospectively observed in melanoma patients receiving chemotherapy after DCs vaccination [69].

In addition to a direct clinical benefit for patients, adjuvant DCs vaccination could also prove useful in improving subsequent treatment response in the event of relapse. These effects were retrospectively observed with administration of ipilimumab in patients with relapse after adjuvant DCs vaccination for stage III melanoma [164].

The full nature of the estimated beneficial effects of DCs vaccination is much more complex than any immune monitoring or molecular biology tool at this stage [165]. Earlier DCs vaccination in the course of the disease is more beneficial due to less tumor-induced immunosuppression, since it more frequently induces tumor-specific immune responses. Therefore, DCs-based immunotherapy might be most suitable in the adjuvant setting, and their additive effects should be considered inside the cancer therapeutic landscape.

## 7. Conclusions and Future Prospects

The hypothesis of cancer immunotherapy was first demonstrated by Coley and colleagues. The key concept of cancer immunotherapy is that the immune system can be manipulated to achieve cancer control and, ideally, therapy. Coley demonstrates this statement by using a blend of bacterial toxins to treat patients with inoperable sarcomas [166]. The antitumor activity of this approach is attributable to the activation of the immune system, largely DCs, which in turn acquires the ability to kill tumor cells. DCs, as key activators of the adaptive immune response, are expected to play a central role in inducing antitumor immune responses. Faced with these data, it became intuitive to make use of DCs potential to induce anticancer responses in cancer patients.

The over 200 clinical trials testing DCs vaccines have shown that DCs are highly immunogenic and safe, often able to activate an antitumor immune response. Some trials have shown that DCs can induce a durable tumor regression and clinical response in treated patients. However, despite several positive data, Sipuleucel-T immunotherapy remains the only therapy to obtain FDA approval to date [11].

DCs research remains critical and DCs will continue to be tested as cancer vaccines in new stages of cancer (earlier stages), new tumors and new combinations.

The synergy between cancer vaccines and conventional therapy (chemotherapy and radiation) has demonstrated a potential role for immunotherapy in multi-modal treatment paradigms. With an improved understanding of tumor biology, checkpoint biology, and immune evasion, we will be able to time and deliver therapy to maximize tumor reactions, promote in situ antitumor immune responses, and enhance patient outcomes.

Combination agents, useful for improving DCs activity, should ideally (1) reduce tumor mass, as high disease burdens harness the clinical efficacy of DCs-schedules; (2) not impair the immune system of the host; (3) release the brakes of ongoing immune response; and (4) synergize directly or indirectly with DCs functions.

The timing of these combinatorial approaches should be carefully considered, as it could affect the efficacy of combination therapies. In addition, determining the optimal combination of therapies likely depends on multiple factors including the patient’s condition, tumor type, stage and composition of the tumor microenvironment. Therefore, a punctual characterization of individual patients will help to select immunotherapies that most likely will work synergistically with DCs therapy.

Further research on interactions between immunotherapy and other therapies is needed to incorporate cancer vaccines into current standard of care to maximize the antitumor potential of each treatment modality.

In the near future, surgery, cytotoxic therapies and immunotherapy will form a “triple alliance”, which already demonstrates great potential in the treatment of cancer patients. While the potential impact of such regimens is recognized, an optimal combination treatment still has to be established in order to improve clinical outcomes and provide a “true personalized therapy”.

## Figures and Tables

**Table 1:** Adjuvant Dendritic Cell Immunotherapy trials for cancer treatment.

Description	N. of Trials	Disease(s)	Status	Phase
DCs based adjuvant therapy versus standard of care (radio/chemotherapy)	79	24 Brain tumors; 13 Melanoma; 12 Breast Cancer; 6 Liver/colo-rectal cancer; 4 pancreatic cancer; 20 other.	15 Recruiting; 19 Not (yet) recruiting; 38 completed/terminated; 7 Unknown/withdrawn	3 in phase 3; other phase 1, 2
DCs based adjuvant therapy plus ICI	42	11 Melanoma; 3 brain tumor; 4 Gastric cancer; 4 lung cancer; 3 pancreatic cancers; 5 Myeloma/Lymphoma; 12 other	15 Recruiting; 9 Not (yet) recruiting; 12 completed/terminated; 6 Unknown/withdrawn	5 in phase 3/4; other phase 1, 2
DCs based adjuvant therapy plus other adoptive cells therapy	29	6 Brain tumors; 3 Melanoma; 1 lung; 7 epato/gastrict tract cancer; 4 Myeloma/Lymphoma; 8 other	8 Recruiting; 4 Not (yet) recruiting; 5 completed/terminated; 12 Unknown/withdrawn	2 in phase 3; other phase 1, 2

Searching on clinicaltrial.gov (accessed on 5 November 2021) using the keywords “dendritic cells”, “adjuvant therapy” and “cancer” led to 150 results. The majority of trials (79) involve the use of DCs as an adjuvant therapy in combination with the standard of care; 42 trials out of 150 used DCs in addiction to ICI; 29 trials used DCs with other adoptive cells therapies. Most of the trials focus on diseases such as brain tumors (33) and melanoma (27), followed by myeloma/lymphoma (16) breast cancer (12), liver/colon-rectal cancer (six), gastric cancer (11), pancreatic (seven) and lung cancer (five). The majority of trials are in early phase (1, 2) only 10 out of 150 are in phase 3 or 4. The complete list of trials is reported in Supplementary Materials Table S1.

## Data Availability

Not applicable.

## Supplementary Materials

The following are available online at https://www.mdpi.com/article/10.3390/ijms222212339/s1.

## Abbreviations

MHC	Major Histocompatibility Complex
TAA	tumor associated antigens
DAMPS	damage-associated molecular patterns
MDSCs	myeloid-derived suppressor cells
COX2	cyclooxygenase-2
IDO	indoleamine 2,3-dioxygenase
CCL3	C-C motif chemokine ligand 3
CCL21	C-C motif chemokine ligand 21
TILs	tumor infiltrating lymphocytes
CTL	cytotoxic T lymphocytes
NK	natural killer cells
ICI	immune checkpoint inhibitors
CIK	cytokines induced killer cells
