# Supplementary material for: Dendritic Cells and Cancer Immunotherapy: The Adjuvant Effect
Source: Int J Mol Sci. 2021 Nov 15;22(22):12339. doi: 10.3390/ijms222212339 (PMC8620771; doi:10.3390/ijms222212339)
Supplement: Supplementary file 1 [file ijms-22-12339-s001.zip › ijms-1421100-supplementary.pdf]

Table S1. Adjuvant Dendritic Cell Immunotherapy in combination with the standard of care for cancer treatment.

Description	Drug(s)	Disease(s)	Status	Phase
Adjuvant Dendritic Cell Immunotherapy for Pediatric Patients With High-grade Glioma or Diffuse Intrinsic Pontine Glioma	Dendritic cell vaccination + temozolomide-based chemoradiation+- conventional next-line treatment	High Grade Glioma Diffuse Intrinsic Pontine Glioma	Not yet recruiting	1, 2
Adjuvant Dendritic Cell-immunotherapy Plus Temozolomide in Glioblastoma Patients	Dendritic cell vaccine plus temozolomide chemotherapy	Glioblastoma Multiforme of Brain	Recruiting	1, 2
Evaluation of the Effects of Local Granulocyte-Macrophage Colony-Stimulating Factor (GM-CSF) in Adjuvant Administration on Dendritic Cells in Skin of Melanoma Patients and in Sentinel Lymph Nodes: MEL38	GM-CSF-in-adjuvant, Montanide ISA-51	Melanoma	Completed	nd
Dendritic Cell-Based Tumor Vaccine Adjuvant Immunotherapy of Human Glioblastoma Multiforme (WHO Grade IV Gliomas)	Dendritic cells vaccine	Glioblastoma Multiforme	Unknown †	2
Comparison of Dendritic Cells Versus Montanide as Adjuvants in a Melanoma Vaccine	KLH; Peptides; Dendritic Cells	Melanoma	Completed	1
A Phase II Study on Adjuvant Vaccination With Dendritic Cells Loaded With Autologous Tumor Homogenate in Resected Stage IV Rare Cancers.	Autologous DC vaccine Interleukin-2	Head Neck Tumors, Neuroendocrine Tumors, Soft Tissue Sarcoma	Recruiting	2
External Beam Radiation With Intratumoral Injection of Dendritic Cells As Neo-Adjuvant Treatment for Sarcoma	Dendritic Cell (DC) Injections, Radiation therapy, Complete Resection - Surgery for tumor removal	Soft Tissue Sarcoma	Completed	2
Neoantigen Dendritic Cell Vaccine and Nivolumab in HCC and Liver Metastases From CRC	Neoantigen Dendritic Cell Vaccine, Nivolumab	Hepatocellular Carcinoma, Colorectal Cancer	Recruiting	2
Complementary Vaccination With Dendritic Cells Pulsed With Autologous Tumor Lysate in Resected Stage III and IV Melanoma Patients.	Autologous Dendritic Cell vaccine Adjuvant Drug Therapy	Malignant Melanoma	Terminated	2
ADCTA for Adjuvant Immunotherapy in Standard Treatment of Recurrent Glioblastoma Multiforme (GBM)	Autologous Dendritic Cell/Tumor Antigen, ADCTA	Glioblastoma Multiforme	Recruiting	3
HER-2 Pulsed DC Vaccine to Prevent Recurrence of Invasive Breast Cancer	HER-2 pulsed Dendritic Cell Vaccine	Breast Cancer	Active, not recruiting	1
Dendritic Cell Immunotherapy Against Cancer Stem Cells in	Dendritic cell immunization, Adjuvant temozolomide	Glioblastoma	Recruiting	2, 3

Glioblastoma Patients Receiving Standard Therapy				
Th-1 Dendritic Cell Immunotherapy Plus Standard Chemotherapy for Pancreatic Adenocarcinoma	Autologous DC vaccine	Pancreatic Adenocarcinoma	Recruiting	1
Safety & Efficacy of DC Vaccine and TMZ for the Treatment of Newly-diagnosed Glioblastoma After Surgery	Autologous dendritic cells pulsed with multiple neoantigen peptides, Temozolomide adjuvant chemotherapy	Glioblastoma Multiforme of Brain	Not yet recruiting	1
Dendritic Cell Vaccine for Patients With Brain Tumors	Autologous tumor lysate-pulsed DC vaccination, resiquimod, adjuvant polyICLC	Glioma, Glioblastoma , Anaplastic Astrocytoma, Anaplastic Astro-oligodendroglioma	Active, not recruiting	2
Personalized Vaccine With SOC Chemo Followed by Nivo in Pancreatic Cancer	Autologous Dendritic Cell Vaccine Loaded with Personalized Peptides (PEP-DC vaccine)	Pancreatic Adenocarcinoma	Recruiting	1
Melanoma Patients Immunized With Natural DenDritic Cells	nDC vaccination	Melanoma (Skin)	Active, not recruiting	3
Dendritic Cell Based Therapy for Breast Cancer Patients	DC vaccine, aromatase inhibitor	Breast Cancer	Withdrawn	2
A Comparison of Matured Dendritic Cells and Montanide® in Study Subjects With High Risk of Melanoma Recurrence	DC Vaccine, Montanide Vaccine, Poly-ICLC	Melanoma	Active, not recruiting	2
Efficiency of Vaccination With Lysate-loaded Dendritic Cells in Patients With Newly Diagnosed Glioblastoma	Autologous, tumor lysate-loaded, mature dendritic cells (DC), standard therapy	Glioblastoma	Recruiting	2
Dendritic Cell Vaccination With Standard Postoperative Chemoradiation for the Treatment of Adult Glioblastoma: Phase I Clinical Trial	TH-1 Dendritic Cell Immunotherapy	Glioblastoma	Not yet recruiting	1
Vaccine Therapy in Treating Patients With Stage III Non-Small Cell Lung Cancer	Mutant p53 peptide pulsed dendritic cell vaccine, adjuvant therapy	Lung Cancer	Completed	2
Decitabine Followed by a Cancer Antigen Vaccine for Patients With Neuroblastoma and Sarcoma	Autologous dendritic cell vaccine with adjuvant	Neuroblastoma, Ewings Sarcoma, Osteogenic Sarcoma	Completed	1
RENEW: Feasibility of CMV RNA-Pulsed Dendritic Cells Vaccines for the Treatment of Newly Diagnosed Glioblastoma Patients.	Autologous DCs derived from PBMC loaded with RNA encoding the human CMV matrix protein pp65-flLAMP plus GM-CSF	Glioblastoma	Not yet recruiting	1
Vaccination With Autologous Dendritic Cells Loaded With Autologous Tumour Homogenate After Curative Resection for Stage IV Colorectal Cancer.	Autologous dendritic cells loaded with autologous tumour homogenate, IL2, Curative Resection	Stage IV Colorectal Cancer	Recruiting	2

Vaccine Therapy With Either Neoadjuvant or Adjuvant Chemotherapy and Adjuvant Radiation Therapy in Treating Women With p53-Overexpressing Stage III Breast Cancer	Autologous dendritic cell-adenovirus p53 vaccine	Breast Cancer	Completed	1, 2
Dendritic Cells Plus Autologous Tumor RNA in Uveal Melanoma	Autologous Dendritic Cells loaded with autologous Tumor RNA	Uveal Melanoma	Recruiting	3
Cytomegalovirus (CMV) RNA-Pulsed Dendritic Cells for Pediatric Patients and Young Adults With WHO Grade IV Glioma, Recurrent Malignant Glioma, or Recurrent Medulloblastoma	CMV-DCs with GM-CSF, Td (tetanus toxoid)	Glioblastoma, Medulloblastoma Recurrent	Completed	1
Phase II Feasibility Study of Dendritic Cell Vaccination for Newly Diagnosed Glioblastoma Multiforme	Autologous Dendritic Cell, Temozolomide, Radiotherapy	Glioblastoma Multiforme	Completed	2
Study With Dendritic Cell Immunotherapy in Resected Hepatic Metastasis of Colorectal Carcinoma	Dendritic cells vaccine	Colorectal Carcinoma, Hepatic Metastasis	Unknown †	2
Vaccination With Autologous Dendritic Cells Loaded With Autologous Tumour Homogenate in Glioblastoma	Autologous Dendritic Cells (DC) vaccine, Temozolomide	Glioblastoma	Not yet recruiting	2
Melanoma Vaccine in Treating Patients With Stage III Melanoma After Surgery to Remove Lymph Nodes	HLA-A1-binding MAGE-1/MAGE-3 multipeptide-pulsed autologous dendritic cell vaccine, HLA-A2-binding TYR/MART-1/gp100 multipeptide-pulsed autologous dendritic cell vaccine	Melanoma (Skin)	Unknown †	1, 2
DC Vaccination in CML	DC vaccine	Myeloid Leukemia, Chronic	Recruiting	1, 2
Messenger Ribonucleic Acid (mRNA) Transfected Dendritic Cell Vaccination in High Risk Uveal Melanoma Patients	Autologous dendritic cells electroporated with mRNA	Uveal Melanoma	Terminated	1, 2
Vaccine Therapy in Treating Patients With Head and Neck Cancer	Mutant p53 peptide pulsed dendritic cell vaccine, tetanus toxoid helper peptide, adjuvant therapy	Head and Neck Cancer	Completed	1
DC Migration Study for Newly-Diagnosed GBM	Human CMV pp65-LAMP mRNA-pulsed autologous DCs, Unpulsed DCs, tetanus toxoid	Glioblastoma, Astrocytoma, Grade IV Giant Cell Glioblastoma, Glioblastoma Multiforme	Active, not recruiting	2
Vaccine Therapy in Treating Young Patients Who Are Undergoing Surgery for Malignant Glioma	Therapeutic autologous dendritic cells	Brain and Central Nervous System Tumors	Completed	1
Dendritic Cell Based Therapy of Renal Cell Carcinoma	Tumor antigen loaded autologous dendritic cells	Advanced Renal Cell Carcinoma	Completed	1, 2

Dendritic Cell Based Therapy of Malignant Melanoma	Tumor antigen loaded autologous dendritic cells	Advanced Melanoma	Completed	1, 2
Vaccine Therapy and GM-CSF in Treating Patients With Recurrent or Metastatic Melanoma	Autologous tumor cell vaccine, therapeutic autologous dendritic cells, sargramostim	Melanoma (Skin)	Completed	2
Dendritic Cell Based Therapy of Metastatic Breast Cancer	Onco-peptide loaded autologous dendritic cells	Metastatic Breast Cancer	Terminated	1, 2
Immunogenicity and Safety of DCs in Breast Cancer	Dendritic cells	Breast Cancer Female	Completed	1, 2
Dendritic Cell-based Immunotherapy in Mesothelioma	Tumor lysate-loaded autologous dendritic cells	Malignant Pleural Mesothelioma	Completed	1
Dendritic Cell/Myeloma Fusion Vaccine for Multiple Myeloma (BMT CTN 1401)	Melphalan	Multiple Myeloma	Active, not recruiting	2
Carcinoembryonic Antigen-loaded Dendritic Cells in Advanced Colorectal Cancer Patients	CEA-loaded dendritic cell vaccine	Colorectal Cancer, Liver Metastases	Completed	1, 2
Efficacy of Dendritic Cell Therapy for Myeloid Leukemia and Myeloma	Biological: dendritic cell vaccination (active specific immunotherapy)	Acute Myeloid Leukemia, Chronic Myeloid Leukemia, Multiple Myeloma	Unknown †	2
Dendritic Cell-based Immunotherapy Combined With Low-dose Cyclophosphamide in Patients With Malignant Mesothelioma	DC + CTX	Malignant (Pleural) Mesothelioma	Completed	1
Dendritic Cells in Lung Cancer	Biological: Allogeneic Tumour Lysate (MelCancerVac)	Non Small Cell Lung Cancer	Completed	2
Cellular Immunotherapy for Patients With High Risk Myelodysplastic Syndromes and Acute Myeloid Leukemia	Autologous dendritic cells electroporated with WT1 mRNA	Myelodysplastic Syndromes, Acute Myeloid Leukemia	Active, not recruiting	1, 2
Immunotherapy Targeted Against Cytomegalovirus in Patients With Newly-Diagnosed WHO Grade IV Unmethylated Glioma	Human CMV pp65-LAMP mRNA-pulsed autologous DCs containing GM CSF, Temozolomide, Tetanus-Diphtheria Toxoid (Td)	Glioblastoma	Recruiting	2
Interferon-gamma or Aldesleukin and Vaccine Therapy in Treating Patients With Multiple Myeloma	Biological: idiotype-pulsed autologous dendritic cell vaccine APC8020, aldesleukin, recombinant interferon gamma	Multiple Myeloma and Plasma Cell Neoplasm	Completed	2
Vaccination of Melanoma Patients With Dendritic Cells Loaded With Allogeneic Apoptotic-Necrotic Melanoma Cells	DC/Apo-Nec	Melanoma	Completed	1
DC Migration Study to Evaluate TReg Depletion In GBM Patients With and Without Varlilumab	Human CMV pp65-LAMP mRNA-pulsed autologous DCs, Temozolomide, Varlilumab	Glioblastoma	Recruiting	2
Autologous Dendritic Cells Loaded With Autologous Tumor Associated Antigens for Treatment of Newly Diagnosed Glioblastoma	AV-GBM-1	Newly Diagnosed Glioblastoma	Active, not recruiting	2

Autologous Dendritic Cells Loaded With Autologous Tumor Associated Antigens for Treatment of Advanced Epithelial Ovarian Carcinomas	AVOVA-1	Stage III Ovarian Carcinoma, Stage IV Ovarian Carcinoma, Fallopian Tube Carcinoma, Primary Peritoneal Carcinoma	Active, not recruiting	2
Administration of Autologous Dendritic Cells (DCs) Infected With an Adenovirus Expressing Her-2	CD34+ derived DCs	Breast Neoplasms	Completed	1
Dendritic Cell Cancer Vaccine for High-grade Glioma	Trivax, Temozolomide, Surgery, Radiotherapy	Glioblastoma Multiforme	Completed	2
Phase IIB TL + YCWP + DC in Melanoma	TLPLDC	Melanoma	Active, not recruiting	2
myDC/pDC in Stage III Melanoma Patients	myDC vaccination, pDC vaccination, combined myDC/pDC vaccination	Melanoma	Active, not recruiting	1, 2
Personalized DC Vaccine for Postoperative Cancer	DC vaccine subcutaneous administration	Gastric Cancer, Hepatocellular Carcinoma, Non-Small-Cell Lung Cancer, Colon Rectal Cancer	Recruiting	1
Immunotherapy Combined With Capecitabine Versus Capecitabine Monotherapy in Advanced Breast Cancer	DC-CIK Immunotherapy, Capecitabine Monotherapy	Breast Cancer	Active, not recruiting	2
Dendritic Cell Vaccination in Patients With Lynch Syndrome or Colorectal Cancer With MSI	DC vaccination	Colorectal Cancer	Active, not recruiting	1 2
Dendritic Cell Vaccine and Chemotherapy for Patients With Pancreatic Cancer	DC Vaccine + Standard of Care Chemotherapy	Pancreatic Cancer	Terminated	1
Paclitaxel, Cyclophosphamide, and Doxorubicin Followed by Autologous Dendritic Cells and Surgery With or Without Radiation Therapy and/or Hormone Therapy in Treating Women With Stage II or Stage III Breast Cancer	Therapeutic autologous dendritic cells, aromatase inhibition therapy	Breast Cancer	Completed	2
HER2 Directed Dendritic Cell Vaccine During Neoadjuvant Therapy of HER2+Breast Cancer	Dendritic Cell Vaccine (DC1), Neoadjuvant Chemotherapy, Curative Surgery	Breast Cancer	Recruiting	Early 1
Basiliximab in Treating Patients With Newly Diagnosed Glioblastoma Multiforme Undergoing Targeted Immunotherapy and Temozolomide-Caused Lymphopenia	RNA-loaded dendritic cell vaccine, basiliximab	Malignant Neoplasms Brain	Completed	1

aDC1 Vaccine + Chemokine Modulatory Regimen (CKM) as Adjuvant Treatment of Peritoneal Surface Malignancies	DC vaccine, Celecoxib, Interferon Alfa-2b, rintatolimod	Malignant Neoplasm of Pancreas Metastatic to Peritoneal Surface, Malignant Peritoneal Mesothelioma, Peritoneal Carcinomatosis	Completed	1, 2
Wilm's Tumor 1 Protein Vaccine to Treat Cancers of the Blood	WT1 Peptide-Pulsed Dendritic Cells, Donor Lymphocytes, IL-4	Leukemia: Acute Myelogenous (AML), Acute Lymphocytic (ALL), , Chronic Myelogenous (CML)	Completed	1, 2
Personalized Vaccine for Cancer Immunotherapy	Peptide pulsed Dendritic cell	Breast Cancer Female	Completed	1
Vaccine Therapy in Treating Patients With Metastatic Melanoma	Therapeutic autologous dendritic cells, filgrastim	Melanoma (Skin)	Unknown †	1
Vaccine Therapy in Treating Patients Who Are Undergoing Surgery for Stage IB, Stage II, or Stage IIIA Non-Small Cell Lung Cancer	Therapeutic autologous dendritic cells, autologous tumor cell vaccine, adjuvant therapy	Lung Cancer	Terminated	1
Vaccination With Dendritic Cells Loaded With Brain Tumor Stem Cells for Progressive Malignant Brain Tumor	Dendritic Cells, Imiquimod	Brain Tumor, Glioblastoma, Medulloblastoma	Completed	1
Dendritic Cell Vaccine With Imiquimod for Patients With Malignant Glioma	Biological: Dendritic Cell Vaccine in combination with Imiquimod cream	Malignant Glioma	Completed	1
Dendritic Cell (DC) Vaccine for Malignant Glioma and Glioblastoma	Dendritic Cell Vaccine, Tumor Lysate, Imiquimod	Malignant Glioma, Glioblastoma Multiforme, Anaplastic Astrocytoma	Active, not recruiting	1
Dendritic Cell Vaccine Therapy With In Situ Maturation in Pediatric Brain Tumors	Dendritic Cell Vaccine, Tumor Lysate, Imiquimod	Glioma, Brain Cancer, Brain Tumor	Terminated	1
Vaccination of Patients With Ovarian Cancer With Dendritic Cell/Tumor Fusions With Granulocyte Macrophage Colony-stimulating Factor (GM-CSF) and Imiquimod	Dendritic Cell/Tumor Fusion Vaccine, GM-CSF, imiquimod	Ovarian Cancer, Primary Peritoneal Cancer, Fallopian Tube Cancer	Active, not recruiting	2
Vaccination of Patients With Breast Cancer With Dendritic Cell/Tumor Fusions and IL-12	DC, Interleukin-12	Breast Cancer	Terminated	1, 2
Adjuvant Dendritic Cell Immunotherapy in combination with Immune Checkpoint Inhibitors				

Description	Drug(s)	Disease(s)	Status	Phase
Multi-center Phase I/IIa Trial of an Autologous Tumor Lysate (TL) + Yeast Cell Wall Particles (YCWP) + Dendritic Cells (DC) Vaccine in Addition to Standard of Care Checkpoint Inhibitor of Choice in Metastatic Melanoma Patients With Measurable Disease.	TLPLDC Vaccine	Metastatic Melanoma	Completed	1, 2
Safety of AV-MEL-1 With Anti-PD-1 Therapy in Metastatic Melanoma	Drug: AV-MEL-1	Metastatic Melanoma	Recruiting	1
Dendritic Cell Vaccines Against Her2/Her3, Cytokine Modulation Regimen, and Pembrolizumab for the Treatment of Brain Metastasis From Triple Negative Breast Cancer or HER2+ Breast Cancer	Anti-HER2/HER3 Dendritic Cell Vaccine<br>Celecoxib<br>Pembrolizumab	Stage IV Breast Cancer, Metastatic Malignant Brain Neoplasm, Metastatic Triple-Negative Breast Carcinoma, Stage IV Breast Cancer	Not yet recruiting	2
Phase I Clinical Trial of Cryoimmunotherapy Against Prostate Cancer	Dendritic cell based cryoimmunotherapy<br>Cyclophosphamide<br>ipilimumab	Prostate Cancer	Completed	1
A Study to Evaluate the Safety and Effectiveness of ILIxadencel Administered Into Tumors in Combination With Checkpoint Inhibitor (CPI) in Patients With ADvanced Cancer	Ilixadencel<br>Pembrolizumab	Carcinoma, Squamous Cell of Head and Neck, Gastric Adenocarcinoma, Gastroesophageal Junction Adenocarcinoma, Non-small Cell Lung Cancer	Active, not recruiting	1, 2
Atezolizumab Plus Tivozanib in Immunologically Cold Tumor Types	Atezolizumab<br>Tivozanib	Bile Duct Cancer<br>Gall Bladder Cancer<br>Breast Cancer	Recruiting	1, 2
Study of SQZ-PBMC-HPV in Patients With HPV16+ Recurrent, Locally Advanced or Metastatic Solid Tumors	SQZ-PBMC-HPV<br>Atezolizumab<br>Ipilimumab<br>Nivolumab	Adult Solid Tumor	Recruiting	1
The Combination of Anti-PD-1 With Radiotherapy in Previously Untreated Metastatic Melanoma	The combination of radiotherapy with anti-PD1 (pembrolizumab or nivolumab)	Melanoma	Recruiting	2
Trial of SBRT With Concurrent Ipilimumab in Metastatic Melanoma	Radiation: Stereotactic body radiotherapy<br>Ipilimumab	Melanoma	Completed	1
Trial to Evaluate Safety and Efficacy of Vinorelbine With Metronomic Administration in Combination	Atezolizumab<br>Vinorelbine	Non-small Cell Lung Cancer	Active, not recruiting	2

With Atezolizumab as Second-line Treatment for Patients With Stage IV Non-small Cell Lung Cancer				
Neoantigen Dendritic Cell Vaccine and Nivolumab in HCC and Liver Metastases From CRC	Neoantigen Dendritic Cell Vaccine<br>Nivolumab	Hepatocellular Carcinoma<br>Colorectal Cancer	Recruiting	2
Combination Immunotherapy-Ipilimumab-Nivolumab-Dendritic Cell p53 Vac - Patients With Small Cell Lung Cancer (SCLC)	Nivolumab<br>Ipilimumab<br>Dendritic Cell based p53 Vaccine	Small Cell Lung Cancer<br>Lung Cancer<br>Relapsed Small Cell Lung Cancer	Active, not recruiting	2
Autologous Dendritic Cells Pulsed With Tumor Lysate Antigen Vaccine and Nivolumab in Treating Patients With Recurrent Glioblastoma	autologous dendritic cells pulsed with tumor lysate antigen Vaccine<br>Laboratory Biomarker Analysis<br>Nivolumab	Giant Cell Glioblastoma<br>Gliosarcoma<br>Oligodendroglioma	Withdrawn	2
Tislelizumab Combined With Anlotinib and 2-cycles Irinotecan as Second Line Treatment of SCLC	Anlotinib Tislelizumab Irinotecan	Small Cell Lung Cancer (SCLC)	Recruiting	Not Applicable
Immunostimulating Interstitial Laser Thermotherapy in Malignant Melanoma	Immunostimulating Interstitial Laser Thermotherapy	Melanoma<br>Malignant Melanoma	Terminated	Not Applicable
Immunostimulating Interstitial Laser Thermotherapy in Solid Cancers	Immunostimulating Interstitial Laser Thermotherapy	Neoplasms	Terminated	Not Applicable
Dendritic Cell (DC)/Myeloma Fusions in Combination With Nivolumab in Patients With Relapsed Multiple Myeloma	Nivolumab<br>DC/myeloma fusions/GM-CSF	Multiple Myeloma	Terminated	2
Clinical Trial of SBRT and Systemic Pembrolizumab With or Without Avelumab/Ipilimumab +Dendritic Cells in NSCLC	Avelumab<br>Ipilimumab 5 MG/ML<br>Pembrolizumab 100 MG in 4 ML Injection<br>CD1c (BDCA-1)+ / CD141 (BDCA-3)+ myDC	Non Small Cell Lung Cancer	Recruiting	2
Polarized Dendritic Cell (aDC1) Vaccine, Interferon Alpha-2, Rintatolimod, and Celecoxib for the Treatment of HLA-A2+ Refractory Melanoma	Alpha-type-1 Polarized Dendritic Cells<br>Celecoxib<br>PD-1 Ligand Inhibitor	HLA-A2 Positive Cells Present<br>Refractory Melanoma	Not yet recruiting	2
Intratumoral Injection of Autologous CD1c (BDCA-1)+ myDC, Avelumab, and Ipilimumab Plus Systemic Nivolumab	Intratumoral injection of autologous CD1c (BDCA-1)+ myDC	Solid Tumor<br>Metastases to Soft Tissue	Completed	1
PANFIRE-3 Trial: Assessing Safety and Efficacy of Irreversible Electroporation (IRE) + Nivolumab + CpG for Metastatic Pancreatic Cancer	Irreversible Electroporation (IRE)<br>Nivolumab<br>Toll-Like Receptor 9	Pancreatic Cancer<br>Metastatic Pancreatic Cancer	Recruiting	1
Dendritic Cell Immunotherapy Plus Standard Treatment of Advanced Renal Cell Carcinoma	CMN-001<br>Nivolumab+Ipilimumab<br>Lenvatinib+Everolimus	Advanced Renal Cell Carcinoma	Recruiting	2

Combination Immunotherapy in Rare Cancers Under InvesTigation	Ipilimumab<br>Nivolumab	Advanced Biliary Tract Cancer<br>Neuroendocrine Tumors<br>Female Reproductive System Neoplasm<br>MSI-H Solid Malignant Tumor	Recruiting	2
A Phase II Trial of Ipilimumab and Nivolumab for the Treatment of Rare Cancers	Ipilimumab<br>Nivolumab	Gastrointestinal Cancer<br>Neuroendocrine Tumours<br>Malignant Female Reproductive System Neoplasm	Active, not recruiting	2
Nivolumab and Eribulin in HER2 Negative Metastatic Breast Cancer	Nivolumab	Metastatic Breast Cancer	Active, not recruiting	1, 2
CP-675,206 (CTLA4-Blocking Monoclonal Antibody) Combined With Dendritic Cell Vaccine Therapy in Treating Patients With Stage III or Stage IV Melanoma That Cannot Be Removed With Surgery	maximum tolerated dose of anti-cytotoxic T-lymphocyte-associated antigen-4 monoclonal antibody	Melanoma (Skin)	Completed	1
Electroporation Potentiated Immunotherapy in Cancer	Pembrolizumab<br>Irreversible electroporation	Pancreas Cancer, Metastatic	Recruiting	2
A Safety Study of the Pan-immunotherapy in Patients With Unresectable/Metastatic Solid Tumors or Lymphomas	Manganese Chloride<br>Anti-PD-1 antibody<br>Combination Product: Systemic therapy	Solid Tumor<br>Lymphoma	Unknown †	1
An Open-label, Phase I/II Study of the Pan-immunotherapy in Patients With Relapsed/Refractory Ovarian Cancer	Manganese Chloride<br>nab-paclitaxel<br>Platinum chemotherapy<br>Sintilimab	Ovarian Cancer	Recruiting	1, 2
Gene-Modified T Cells, Vaccine Therapy, and Nivolumab in Treating Patients With Stage IV or Locally Advanced Solid Tumors Expressing NY-ESO-1	Aldesleukin<br>Cyclophosphamide<br>Fludarabine Phosphate	Adult and Child Solid Neoplasm<br>Metastatic Neoplasm	Completed	1
Immune Modulation Study in Patients With Metastatic Melanoma Treated With Anti-PD1 Monoclonal Antibodies	blood sampling<br>Nivolumab	Metastatic Melanoma	Unknown †	4
Immunization Strategy With Intra-tumoral Injections of Pexa-Vec With Ipilimumab in Metastatic / Advanced Solid Tumors.	Pexa-Vec<br>Ipilimumab	Metastatic Tumor<br>Advanced Tumor	Recruiting	1
Phase 1 Trial of Ipilimumab and GVAX in Patients With Metastatic Castration-resistant Prostate Cancer	GVAX and ipilimumab	Prostate Cancer	Terminated	1

Tremelimumab and CP-870,893 in Patients With Metastatic Melanoma	CD40 agonist monoclonal antibody CP-870,893<br>tremelimumab<br>laboratory biomarker analysis	Recurrent Melanoma<br>Stage IV Melanoma	Completed	1
A Phase I/II Study of the Pan-immunotherapy in Patients With Local Advanced/Metastatic BTC	Manganese Chloride<br>nab-paclitaxel<br>Gemcitabine<br>anti-PD-1 antibody	Biliary Tract Cancer (BTC)	Unknown †	1, 2
An Open-label, Phase I/II Study of the Pan-immunotherapy in Patients With Local Advanced/Metastatic Pancreatic Cancer	Manganese Chloride<br>nab-paclitaxel<br>Gemcitabine<br>anti-PD-1 antibody	Pancreatic Cancer	Recruiting	1, 2
Nivolumab in Treating Patients With Relapsed or Refractory Peripheral T-cell Lymphoma	Nivolumab	Blastic Plasmacytoid Dendritic Cell Neoplasm<br>Hepatosplenic T-Cell Lymphoma<br>HTLV-1 Infection	Terminated<br>Has Results	2
Abatacept in earLy Onset Polymyalgia Rheumatica: Study ALORS	Abatacept<br>Placebos	Polymyalgia Rheumatica	Unknown †	3
Ipilimumab and All-Trans Retinoic Acid Combination Treatment of Advanced Melanoma	VESANOID<br>Ipilimumab	Melanoma	Active, not recruiting<br>Has Results	3
Nivolumab, BMS-936558 in Combination With Relatlimab, BMS-986016 in Patients With Metastatic Melanoma Naïve to Prior Immunotherapy in the Metastatic Setting	Relatlimab<br>Nivolumab<br>Relatlimab + Nivolumab	Melanoma	Recruiting	3
Effect on Orencia (Abatacept) on the Apoptosis of T Cells, B Cells and APC Cells in Rheumatoid Arthritis	Group 1 or Orencia treated group<br>Group 2 (DMARDS treated group)	Apoptotic DNA Damage<br>Rheumatoid Arthritis<br>T-cell Lymphocytosis	Withdrawn	4
Single Agent and Combined Inhibition After Allogeneic Stem Cell Transplant	Nivolumab<br>Ipilimumab	Acute Myeloid Leukemia and Myelodysplastic Syndrome	Active, not recruiting	1

Adjuvant Dendritic Cell Immunotherapy in combination with other Adoptive Cells Therapies for cancer treatment.

**Description**	**Drug(s)**	**Disease(s)**	**Status**	**Phase**
PEACH TRIAL- Precision Medicine and Adoptive Cellular Therapy	Tumor-specific ex vivo expanded autologous lymphocyte transfer (TTRNA-xALT)	Neuroblastoma<br>Diffuse Intrinsic Pontine Glioma	Recruiting	1
T Cell Transfer With or Without Dendritic Cell Vaccination in Patients With Melanoma	Cyclophosphamide<br>Fludarabine<br>T cells	Melanoma	Recruiting	1

Adoptive Cellular Therapy in Pediatric Patients With High-grade Gliomas	TTRNA-DC vaccines with GM-CSF<br>Dose-intensified TMZ<br>Autologous Hematopoietic Stem cells (HSCs)	Malignant Glioma<br>High Grade Glioma	Recruiting	1
Lymphodepletion Plus Adoptive Cell Transfer With or Without Dendritic Cell Immunization in Patients With Metastatic Melanoma	Dendritic Cell Immunization<br>Cyclophosphamide<br>Fludarabine	Melanoma	Active, not recruiting	2
Gene-Modified T Cells, Vaccine Therapy, and Ipilimumab in Treating Patients With Locally Advanced or Metastatic Malignancies	cyclophosphamide<br>fludarabine phosphate<br>NY-ESO-1 reactive TCR retroviral vector transduced autologous PBL	Unspecified Adult Solid Tumor, Protocol Specific	Terminated	1
Autologous SCT Followed by Dendritic Cell p53 Vaccination in Patients With Limited Stage Small Cell Lung Cancer	Combined adenovirus vectored p53 tranfected dedritic cell vaccine and ex vivo expanded T-lymphocytes	Small Cell Lung Cancer	Terminated	2
Gene-Modified T Cells, Vaccine Therapy, and Nivolumab in Treating Patients With Stage IV or Locally Advanced Solid Tumors Expressing NY-ESO-1	Aldesleukin<br>Cyclophosphamide<br>Fludarabine Phosphate	Adult Solid Neoplasm<br>Childhood Solid Neoplasm<br>Metastatic Neoplasm	Completed	1
Adoptive Cell Therapy Plus Chemotherapy and Radiation After Surgery in Treating Patients With Colorectal Cancer	DC-CIK<br>Radiotherapy<br>Chemotherapy	Colorectal Cancer<br>Neoplasms<br>Intestinal Neoplasms	Unknown †	2
Study of Gene Modified Immune Cells in Patients With Advanced Melanoma	F5 TCR transgenic cells and MART-1 peptide pulsed dendritic cells<br>non-myeloablative conditioning chemotherapy	Metastatic Melanoma	Completed	2
Flotetuzumab for Relapsed Acute Myeloid Leukemia (AML) and Myelodysplastic Syndrome (MDS) Following Allogeneic Hematopoietic Cell Transplantation (Allo-HCT)	MGD006<br>Donor lymphocyte infusion	Relapsed Acute Myeloid Leukemia	Recruiting	2
Study of Chemotherapy With Adoptive Cellular Therapy With DC-CIK Cells in Triple Negative Breast Cancer Patients	DC-CIK<br>Standard chemotherapy	Breast Neoplasms<br>Neoplasm Metastasis	Completed	nd
Vaccine Immunotherapy for Recurrent Medulloblastoma and Primitive Neuroectodermal Tumor	TTRNA-xALT<br>TTRNA-DCs	Medulloblastoma<br>Neuroectodermal Tumor	Active, not recruiting	1, 2
Brain Stem Gliomas Treated With Adoptive Cellular Therapy During Focal Radiotherapy Recovery Alone or With Dose-intensified Temozolomide (Phase I)	TTRNA-DC vaccines with GM-CSF<br>TTRNA-xALT<br>Cyclophosphamide + Fludarabine Lymphodepletive Conditioning	Diffuse Intrinsic Pontine Glioma (DIPG)<br>Brain Stem Glioma	Recruiting	1
Immunotherapy Using Precision T Cells Specific to Personalized Neo-antigen for the Treatment of Advanced Malignant Tumor of Biliary Tract	Gemcitabine<br>Dendritic cell-precision T cell for neo-antigen combined with gemcitabine treatment	Advanced Biliary Tract Malignant Tumor	Unknown †	1, 2

Gene and Vaccine Therapy in Treating Patients With Advanced Malignancies	aldesleukin<br>fludarabine phosphate<br>cyclophosphamide	Malignant Neoplasm	Active, not recruiting	2
Pilot Study of T-APCs Following CAR T Cell Immunotherapy for CD19+ Leukemia	T-cell Antigen Presenting Cells expressing truncated CD19 (T-APC)	CD 19+ Acute Leukemia	Recruiting	1
Concurrent Chemoradiation With or Without DC-CIK Immunotherapy in Treating Locally Advanced Esophageal Cancer	concurrent chemoradiotherapy plus DC-CIK immunotherapy<br>Concurrent chemoradiation only	Esophageal Carcinoma	Recruiting	Not Applicab le
Immunotherapy Using Pluripotent Killer-Programmed Cell Death 1 (PIK-PD-1) Cells for the Treatment of Advanced Hepatocellular Carcinoma	PIK-PD-1 cells<br>DC-PMAT	Advanced Hepatocellular Carcinoma	Unknown †	1, 2
Evaluation of Recovery From Drug-Induced Lymphopenia Using Cytomegalovirus-specific T-cell Adoptive Transfer	CMV-ALT + CMV-DCs<br>CMV-ALT + Saline	Glioblastoma	Completed	1
Immunotherapy Using Pluripotent Killer-Human Epidermal Growth Factor Receptor-2 (PIK-HER2) Cells for the Treatment of Advanced Gastric Cancer With Liver Metastasis	PIK-HER2<br>DC-PMAT	Liver Metastasis<br>Gastric Cancer	Unknown †	1, 2
Specialized Immune Cells (nCTLs) and a Vaccine (Alpha-type-1 Polarized Dendritic Cells) in Treating Patients With Stage II-IV Ovarian, Fallopian Tube, or Primary Peritoneal Cancer	Alpha-type-1 Polarized Dendritic Cells<br>Autologous Natural Killer Cell-like CTLs	Stage II Fallopian Tube Cancer AJCC v8<br>Stage II Ovarian Cancer AJCC v8<br>Stage II Primary Peritoneal Cancer AJCC v8	Not yet recruiting	1, 2
Proteome-based Personalized Immunotherapy of Glioblastoma	Dendritic vaccine, allogeneic hematopoietic stem cells, cytotoxic lymphocytes<br>Dendritic vaccine, autologous hematopoietic stem cells, cytotoxic lymphocytes	Glioblastoma	Unknown †	1, 2
Proteome-based Immunotherapy of Brain Metastases From Breast Cancer	Dendritic vaccine, allogeneic hematopoietic stem cells, cytotoxic lymphocytes<br>Dendritic vaccine, autologous hematopoietic stem cells, cytotoxic lymphocytes	Neoplasm Metastasis	Unknown †	2, 3
Proteome-based Immunotherapy of Lung Cancer Brain Metastases	Dendritic vaccine, allogeneic hematopoietic stem cells, cytotoxic lymphocytes<br>Dendritic vaccine, autologous hematopoietic stem cells, cytotoxic lymphocytes	Neoplasm Metastasis	Unknown †	2, 3
HA-1 T TCR T Cell Immunotherapy for the Treatment of Patients With Relapsed or Refractory Acute Leukemia After Donor Stem Cell Transplant	CD8+ and CD4+ Donor Memory T-cells-expressing HA1-Specific TCR<br>Fludarabine<br>Laboratory Biomarker Analysis	Juvenile Myelomonocytic Leukemia<br>Recurrent Acute Biphenotypic Leukemia	Recruiting	1

		Recurrent Acute Undifferentiated Leukemia		
The Adoptive Immunotherapy for Solid Tumors Using Modified Autologous Cytokine-induced Killer Cells	cytokine induced killer cells	Cholangiocarcin oma	Unknown †	1, 2
DC-CIK In Combination With Chemotherapy (Gio / Oxaliplatin or Cisplatin) Versus First-line Chemotherapy for Locally Advanced Unresectable or Metastatic Gastric Adenocarcinoma Randomized Controlled Phase II Clinical Study of Treatment	Autologous dendritic cells co-cultured with cytokine-induced killer cells, Gimeracil and Oteracil Porassium Capsules, Oxaliplatin	Gastric Cancer	Unknown †	2
Clinical Study of DC Plus CIK for Patients With Relapse Acute Leukemia After Allo-HSCT	Genetically modified DCs plus CIK cells, Donor leukocyte infusions (DLI)	Acute Leukemia	Unknown †	1, 2
